# Exploring the Association Between Heart Rate Variability and Intracranial Atherosclerosis in Middle-Aged or over Community-Dwelling Adults

**DOI:** 10.3390/diagnostics15212731

**Published:** 2025-10-28

**Authors:** Yangyang Cheng, Lihua Lai, Jieqi Luo, Michael Tin Cheung Ying

**Affiliations:** 1 Department of Health Technology and Informatics, The Hong Kong Polytechnic University, Hong Kong; 22037285r@connect.polyu.hk; 2 Department of Radiology, Shenzhen Third People’s Hospital, Shenzhen 518112, China; lailihua7@163.com (L.L.); jackieluo1984@163.com (J.L.)

**Keywords:** intracranial atherosclerosis, heart rate variability, HR-MRI

## Abstract

**Background/Objectives**: Heart rate variability (HRV) is associated with the risk of vascular events. However, the predictive value of HRV for the presence of intracranial atherosclerosis (ICAS) is unclear. This study aimed to investigate the relationship between daytime HRV measured by 3 min ECG monitoring and ICAS identified by high-resolution magnetic resonance imaging (HR-MRI). **Methods**: A total of 272 adults (mean age, 63.4 ± 6.8; 43% male) were recruited from November 2022 to December 2024. A series of cardiac function parameters is automatically generated through a 3 min analysis by the electrocardiographic dispersion mapping (ECG-DM) software, including heart rate variability and myocardial ischemic metabolic impairment. HRV was assessed as the standard deviation of normal-to-normal intervals (SDNN), which was categorized into tertiles for data analysis. Myocardial micro-alteration index (MMI, %) was used as an indicator of ischemia, reflecting myocardial abnormalities at the metabolic level. Atrial and ventricular myocardial oxygenation deficits were directly visualized in a color-coded scatter plot, with different colors indicating the severity of pathological changes. On HR-MRI intracranial artery wall scanning, the prevalence of ICAS was assessed in middle cerebral arteries (MCAs), vertebral arteries (VAs), and basilar arteries (BAs), and the associated plaque characteristics (eccentricity, thickening patterns, remodeling index, and surface morphology) were evaluated. **Results**: Among the subjects, 209 arterial lesions caused by ICAS were detected in 152 subjects (56%), including MCAs (105/544), VAs (68/526), and BAs (36/272). Ninety-four subjects (94/272) with significant HRV deviation had ICAS (*p* = 0.040). Furthermore, subjects with ICAS were more likely to present with atrial hypoxia (*p* = 0.030) compared to those without ICAS. In multivariate analyses, lower standard deviation of normal-to-normal intervals (SDNN, odds ratio, OR = 1.55, 95% CI 1.10–2.18, *p* = 0.012) and atrial deviation (OR = 1.85, 95% CI 1.10–3.14, *p* = 0.022) were independently associated with the presence of ICAS. **Conclusions**: Among middle-aged or older adults in a local community, our study suggested that lower HRV and significant atrial hypoxia were independently associated with the presence of ICAS.

## 1. Introduction

Stroke, as a key contributor to adult disability, ranks as the third most common cause of mortality globally. Intracranial atherosclerosis (ICAS) is a leading cause of ischemic stroke with the highest rate of recurrent stroke in Asia [1,2]. Population-based studies have shown that compared to Caucasians (a prevalence of 8%) [3], Asians have a much higher incidence rate of ICAS in stroke-free individuals, ranging from 3.5% to 45% [4,5,6], probably due to differences in traditional cerebrovascular risk factors such as aging, hypertension, diabetes and smoking, imaging techniques, and definitions of ICAS [7,8,9,10,11]. Recently, a growing body of research has focused on the quest for novel risk factors as markers for the early identification of ICAS. Autonomic nervous system (ANS) dysfunction, recently acknowledged as a novel risk factor, has been associated with increased risk of cerebrovascular diseases [12]. The main proposed mechanisms involve the modulatory effects of ANS on cerebral circulatory autoregulation and blood pressure regulation [13,14,15].

Heart rate variability (HRV) is commonly deemed as an ingenious metric of ANS function to monitor the balance of the sympathetic–vagal nervous system [16,17]. Recently, some studies have demonstrated that reduced HRV serves as a valuable marker for stratifying multiple cardiovascular risk factors and is related to cerebrovascular disease [17,18,19,20,21]. As stated by Mäkikallio AM et al., HRV reduction has been strongly associated with cardiovascular mortality in elderly post-stroke survivors [22]. Another prior study has shown an impactful association between lower nighttime HRV and a higher risk of incident ischemic stroke [23]. Despite some studies indicating that lower HRV is associated with an increased risk of cerebrovascular disease, no research has yet explored the potential correlation between daytime HRV and intracranial atherosclerosis, along with other distinct plaque imaging characteristics in a sub-healthy population.

Therefore, we hypothesized that short-term daytime HRV could serve as an early indicator of an increased risk for intracranial atherosclerotic lesions. This study aimed to explore the association between HRV, as measured by 3 min ECG monitoring, and ICAS, along with distinct atherosclerotic plaque features identified by high-resolution magnetic resonance imaging (HR-MRI). We sought to provide valuable clinical insights for early stratification of high-risk stroke-free populations, better understanding their pathological mechanisms and optimization of prevention strategies.

## 2. Materials and Methods

### 2.1. Subjects and Baseline Characteristics

**Figure 1:**
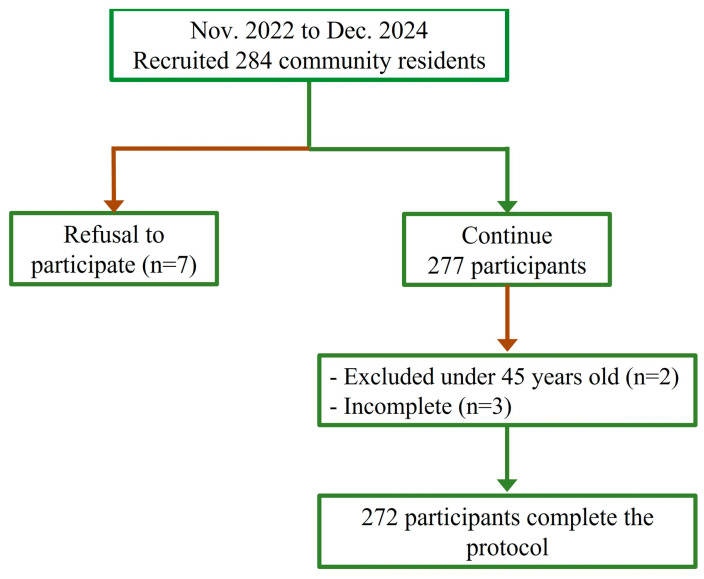
The flowchart shows the subjects’ selection in this study.

This is an observational study conducted from November 2022 to December 2024. Initially, 284 adults aged ≥45 years were consecutively recruited from a local community. The study was approved by the Institutional Review Board of the Hong Kong Polytechnic University (reference number: HSEARS20210720002), and all subjects gave written informed consent. The demographic and clinical information of the subjects was collected from self-reported questionnaires by a trained neurological research assistant, electronic medical records if available, and direct measurements. Vital signs (heart rate, blood pressure, height) and other relevant indices were recorded by a trained research assistant, thereby ensuring an accurate and comprehensive dataset. The inclusion criteria were as follows: (1) subjects aged over 45 years old, free from any prior stroke or transient ischemic attack (TIA); (2) subjects underwent HR-MRI scanning and completed the whole process of the study. The exclusion criteria included the following: (1) any contraindication for MRI; (2) other cerebral vasculopathies, including dissection, vasculitis, aneurysm, or moyamoya disease; (3) history of heart diseases (e.g., heart failure, congenital heart disease, or arrhythmias); (4) poor imaging quality. Figure 1 indicates the subjects’ selection in this study.

Baseline information of all participants was recorded from a questionnaire, including blood pressure, body mass index (BMI), other vascular risk factors (hypertension, diabetes mellitus, hyperlipidaemia, etc.), and medication use.

### 2.2. Cardiac Function Assessment

Electrocardiographic recordings were acquired using a 3 min ECG-DM monitoring system (HeartVue system, KARDi2/4-B, Medical Computer Systems Ltd., Moscow, Russia), consistent with prior studies that used a standard 3 min ECG-DM examination protocol [24,25]. Currently, the new electrocardiographic dispersion mapping (ECG-DM) serves as an innovative “cardiovisor” with higher sampling frequencies to monitor myocardial electric instability and offers more comprehensive and robust data, which is especially important for capturing subclinical myocardium alterations and heart rate micro-alteration during dynamic movements [26]. By segmental analysis in the heart (G1–G9), 3 min ECG-DM offers higher spatial resolution and sensitivity than time-based standard ECG, enabling precise localization of depolarization and repolarization abnormalities in which micro-amplitude changes may precede conventional ECG findings [24]. During the recording, the electrocardiographic parameters including HRV, myocardial micro-alternation index (MMI), atrial/ventricular myocardial hypoxia, and heart rate were automatically recorded by the 3 min ECG-DM and analyzed by the HealthExpress HRV Package system, Moscow, Russia. HRV is a noninvasive procedure that can be performed within a short duration, and it only requires the placement of the four standard ECG limb leads [27]. It reflects the magnitude of RR interval variation over time. Parameters were classified as time and frequency domains that can be obtained through 3 min, 5 min, or 24 h continuous ECG recordings [17]. In the present study, time-domain measurement, defined as the SD of normal-to-normal (NN) intervals (SDNN) which characterizes overall HRV, is categorized into tertiles for HRV assessment. In the regression model, SDNN was analyzed as a binary categorical variable based on the ECG system’s predefined reference range (normal: 17.9–40.1 ms), with in-range values coded as “normal” and out-of-range values coded as “abnormal”. Moreover, three frequency domain parameters were also analyzed, including high frequency power (HF, Hz), low frequency power (LF, Hz), and low/high frequency power ratio (LF/HF). In addition, the overall MMI, expressed as a percentage, serves as an indicator of ischemia which reflects myocardial abnormalities at the metabolic level [24,25]. Myocardial oxygen level deficits are directly visualized in a color-coded scatter diagram, indicating the severity of pathological changes across different segments of the atrial and ventricular myocardium, with green indicating a normal state and red representing ischemic and hypoxic changes [24]. However, this technology’s application is limited in patients with arrhythmia due to insufficient validation data. ECG-DM, therefore, is not a diagnostic test but a nonspecific indicator of myocardial health.

ECG-DM is effective for detecting micro-vibration amplitude and has been shown to identify myocardial electrophysiological abnormalities with a sensitivity of 93% and a specificity of 75% [24]. Dispersion mapping is derived from changes in ECG micro-voltage magnitude, and amplitude shifts are analyzed as signals approach the threshold of myocardial instability. Dispersion characteristics are classified into nine groups (G1-G9), reflecting the magnitude and localization of atrial and ventricular disturbances during depolarization and repolarization. Within the G1–G9 mapping index, results are reported as a myocardial micro-alternation index (0–100%), quantifying deviations or micro-vibratory disturbances. Participants were assessed in a seated resting position. Conductive gel was applied to both wrists and ankles prior to the placement of four leads. A 3 min ECG-DM recording was then acquired, with participants instructed to remain motionless and silent throughout.

### 2.3. MRI Imaging Acquisition

The MRI examinations were performed using a 3.0 T scanner (Siemens Medical Systems, XR Numarism, Germany) with a 64-channel head coil. High-resolution vascular sequences (HR-MRI, a transverse 3D T1-weighted volumetric isotopically reconstructed turbo spin echo acquisition, detailed parameters were as follows: field of view, 53 × 210 × 138 mm^3^; acquired resolution, 0.7 × 0.7 × 0.7 mm^3^; repetition time [TR]/echo time [TE], 900/15 ms; slice thickness, 0.66 mm) were acquired to identify intracranial large-arterial lesions. The standard MRI protocol consisted of 3-dimensional (3D) time-of-flight magnetic resonance angiography (3D TOF-MRA; field of view, 263 × 350 × 350 mm^3^; acquired resolution, 0.5 × 0.5 × 0.3 mm^3^; repetition time/echo time, 20.3/4.3 ms), T2-weighted (field of view, 263 × 350 × 350 mm^3^; repetition time [TR]/echo time [TE], 4100–6000/88 and 99 ms; slice thickness, 4.0 mm), T1-weighted (field of view, 240 × 256 × 167 mm^3^; acquired resolution, 0.8 × 0.8 × 0.8 mm^3^; repetition time [TR]/echo time [TE], 2500/2.22 ms; slice thickness, 0.80 mm), and FLAIR sequences (fluid-attenuated inversion recovery, the parameters were as follows: field of view, 230 × 230 × 173 mm^3^; acquired resolution, 0.9 × 0.9 × 0.9 mm^3^; repetition time [TR]/echo time [TE], 7000/395 ms; slice thickness, 0.90 mm).

### 2.4. Imaging Analysis

MRI images were reviewed on OsiriX DICOM Viewer (Geneva, Switzerland), and quantitative measurements were performed using Vesselmass (Leiden University Medical Center, The Netherlands) [28,29,30,31]. MRI images were independently analyzed by two observers (YY. C and LH. L) who have at least 3 years of experience in MRI image interpretation, and they were all blind to the clinical data of the participants. In the case of disagreement between the two observers, the images were reviewed by a third observer (JQ. L), who has more than ten years of neuroimaging experience, and a consensus agreement was made among the three observers. The adjudicated consensus results were used for the final statistical analyses.

**Figure 2:**
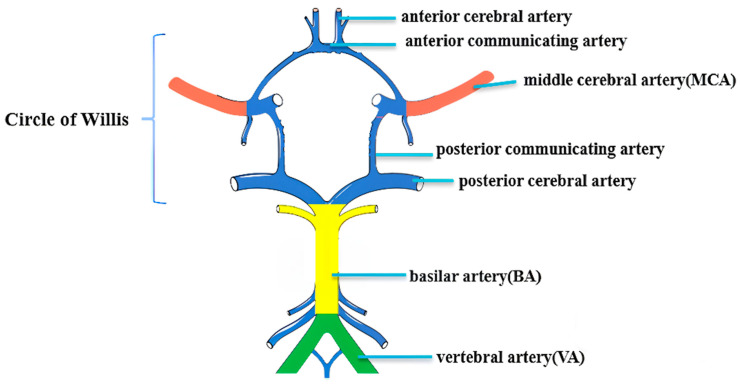
Distribution of intracranial large arteries in the Circle of Willis.

**Figure 3:**
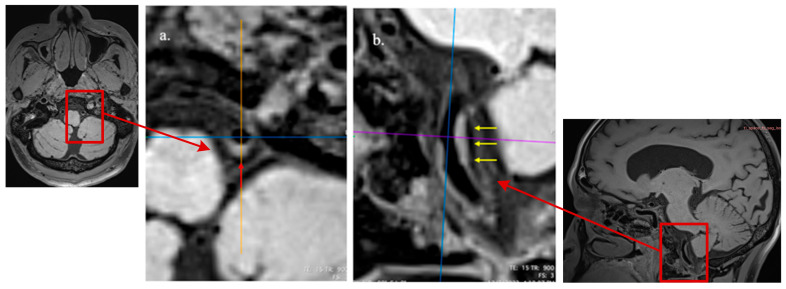
Diffuse and concentric pattern of plaque characteristics at cross-sectional and axial views. HR-VWI reveals mild stenosis of the left vertebral artery, as seen in the reconstructed cross-sectional view ((**a**), red arrow) and axial view ((**b**), yellow arrow).

We analyzed all the plaques involving bilateral M1 segments of middle cerebral arteries (MCAs), the basilar artery (BA), and bilateral intracranial segments of vertebral arteries (VAs) by an intracranial artery illustration on the Circle of Willis (Figure 2) and longitudinal and cross-sectional HR-MRI imaging observations [31,32,33,34] (Figure 3). Based on the method described by previous studies [35,36], for each large artery, only the atherosclerotic lesion with the maximum wall thickness when two or more plaque lesions were identified was analyzed in this study using the nearest plaque-free cross-section proximal to the plaque as the reference site [31,37]. The assessment of the plaque imaging features included plaque burden [31], remodeling index (RI) [38,39], and other morphological patterns, e.g., focal/diffuse thickening patterns, irregular surface, and eccentricity [31,32]. We further assessed the degree of stenosis by a previously described method: (1-lumen area lesion/ lumen area reference) × 100% [31]. In light of the absence of well-defined criteria for precisely evaluating the severity of intracranial arterial stenosis and considering that major intracranial arteries are slightly narrower and have more branches than extracranial arteries, we followed previous reports in our classification. Vessels with stenotic degrees over 25% on HR-MRI were identified on the matched TOF-MRA and classified the stenosis as normal or <25%, 25–49%, ≥50% stenosis [40,41,42].

### 2.5. Statistical Analysis

IBM SPSS version 27.0 (SPSS Inc., Chicago, IL, USA) was used for statistical analysis. All the continuous variables were described as means ± standard deviation (SD) or as medians and interquartile ranges (IQRs) for normally distributed or non-normally distributed data, respectively. Categorical variables were presented as numbers and percentages (%). Fisher’s exact test, Pearson χ^2^, or the Mann–Whitney U test was used to detect the differences among independent groups when appropriate. Tertiles of SDNN were divided into three groups to assess the association between demographic factors and plaque characteristics. One-way ANOVA with post hoc Bonferroni correction or Kruskal–Wallis test with Dunn’s post hoc analysis was applied for three-group comparisons when appropriate. However, we did not apply an additional across-outcome multiplicity correction. To determine the independent association between HRV and the presence of ICAS, univariate and multivariate logistic regressions were utilized after adjusting for confounders, including age, WMH burden, gender, hypertension, BMI, and hyperlipidemia. The inter-rater reliability was assessed by Cohen’s kappa analysis. A two-sided *p* < 0.05 was considered statistically significant.

## 3. Results

### 3.1. Demographic and Clinical Characteristics

In this study, 277 participants were recruited. Of these subjects, two participants did not meet the study’s inclusion criteria, and three participants had incomplete MRI examination or 3 min ECG recording. Finally, 272 participants were included in the study analysis. Baseline characteristics of the participants are shown in Table 1. Overall, the mean age was 63.4 ± 6.8 years, 118 (43.4%) subjects were male, and the mean BMI was 23.7 ± 3.3 kg/m^2^. Participants with ICAS (55.8%) tended to be older (64.1 ± 6.8 years old vs. 62.4 ± 6.7 years old, *p* = 0.041) and had higher systolic blood pressure (132.7 ± 16.3 mmHg vs. 126.4 ± 17.5 mmHg, *p* = 0.003) and greater diastolic blood pressure (81.5 ± 10.1 mmHg vs. 77.8 ± 10.7 mmHg, *p* = 0.005) compared to the non-ICAS group. Participants with ICAS had higher rates of hypertension (38.8% vs. 17.5%, *p* < 0.001) and hyperlipidemia (47.4% vs. 26.7%, *p* < 0.001). Among those with antihypertensive drugs, ICAS had a higher prevalence relative to non-ICAS findings (36.2% vs. 15.1%, *p* < 0.001).

### 3.2. Comparisons of Characteristics of the Electrocardiogram Between Adults with and Without ICAS

**Figure 4:**
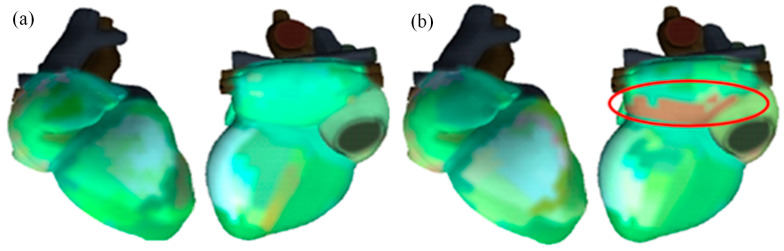
The physiological functional status of the myocardium in different cardiac segments using a color-coded ECG dispersion map. Green indicates a healthy state, as shown in (**a**); yellow represents the normal upper limit, and red indicates myocardial ischemic injury. (**b**) indicates local hypoxia occurring in the left atrial myocardium (the area is shown by an elliptical red outline).

Among 272 participants, 102 (37.5%) showed abnormal electrical activity waveforms with a mean heart rate of 72.2 ± 10.4 bpm. Participants with ICAS were more likely to have atrial myocardium oxygen deficiency impairment when compared to those without ICAS (70.2% vs. 57.5%, *p* = 0.030). Figure 4 indicates the physiological functional status of the myocardium in different cardiac segments using a gradated color-coded image on ECG dispersion mapping. Based on the time-domain SDNN of overall HRV, irregular changes in heartbeat were detected in 94 cases, of which 60 (39.7%) also had ICAS, compared to 28.3% participants without ICAS (*p* = 0.040) (Table 2).

### 3.3. Comparison of Subject Characteristics Across SDNN Tertiles

Participants with lower SDNN values were older (65.0 ± 5.9 years old vs. 63.3 ± 6.6 years old vs. 62.0 ± 7.4, *p* = 0.010) and more likely to have a history of hypertension (34.4% vs. 35.2 vs. 18.9%, *p* = 0.026), compared with the highest SDNN group. Moreover, participants with the lowest SDNN deviation were more prone to be affected by ICAS (55.5% vs. 48.3% vs. 49.4%, *p* = 0.032) compared to participants with higher SDNN values. However, no significant correlation was found between abnormal SDNN and the distinct imaging features of atherosclerotic plaque patterns (all *p* > 0.05) (Table 3). Hence, the comparison among SDNN tertile groups revealed that participants with lower SDNN values were older, more likely to have a history of hypertension, and frequently had higher prevalence of ICAS compared with the highest SDNN value group.

### 3.4. Association Between Heart Rate Deviation and the Presence of ICAS

In the binary regression analysis, we found that abnormal SDNN and atrial myocardial hypoxia are independently associated with the presence of ICAS, with this risk being attributable to lower SDNN values. Finally, univariate logistic regression analysis showed that lower SDNN was an important quantitative global marker of HRV (OR, 1.40; 95% CI, 1.01–1.94; *p* = 0.044) and atrial deviation was closely associated with the presence of ICAS (OR, 1.74; 95% CI, 1.05–2.87; *p* = 0.031), as shown in Table 4. After covariate adjustment for age, gender, hypertension, and hyperlipidemia, the presence of ICAS was significantly associated with lower SDNN (OR, 1.55; 95% CI, 1.10–2.18; *p* = 0.012) and atrial myocardial hypoxia (OR, 1.85; 95% CI, 1.10–3.14; *p* = 0.022).

### 3.5. Inter-Rater Reliability of Assessments on Characteristics of ICAS Patterns

Inter-observer agreement was assessed by randomly selecting 227 subjects, as shown in Table 5, according to the initially independent, blinded readings of the two observers. The inter-observer agreements of the two raters in analyzing the characteristics of ICAS with other distinct plaque imaging morphological features were good. Cohen’s kappa of ICAS presence was 0.86 (95% CI, 0.793–0.926, *p* < 0.001). Kappa of plaque eccentricity was 0.77 (95% CI, 0.692–0.847, *p* < 0.001), plaque irregularity was 0.82 (95% CI, 0.727–0.912, *p* < 0.001), and diffuse thickening was 0.71 (95% CI, 0.631–0.797, *p* < 0.001). Disagreements between the two observers were resolved by a third senior reader, and the adjudicated consensus results were used in the primary analyses.

## 4. Discussion

This study demonstrates, for the first time, the potential relationship between cardiac electrophysiological function and ICAS in relatively healthy stroke-free middle-aged/older Chinese adults. We found evidence of a higher risk of ICAS occurrence among participants with lower HRV and higher atrial myocardial hypoxia, after controlling for confounding variables. These results were consistent across most, but not all, of the time-domain HRV measures. Our findings provide the first evidence suggesting that lower HRV may serve as a valuable indicator of cardiac dysfunction and is associated with the presence of intracranial atherosclerosis. These results also shed light on the underlying pathophysiological mechanisms associated with this relationship, which may not be fully attributable to traditional vascular risk pathways.

HRV, as a physiological metric, describes a series of automatic calculations for psychophysiological responses between consecutive heartbeats [43,44]. A lower HRV is proven to be linked to either increased parasympathetic activity or decreased sympathetic activity and is correlated with multiple vascular events and an elevated risk of all-cause mortality [18,23]. However, some other studies failed to identify an association between HRV and subclinical cerebrovascular disease [45]. Till now, the association of HRV in cerebrovascular diseases remains unclear and conflicting [42,45,46,47]. In the Multi-Ethnic Study of Atherosclerosis, Whelton et al. indicated that higher nocturnal resting heart rate is associated with increased carotid and aortic arterial stiffness [48]. The inconsistency might be caused by different study designs and populations. In line with previous reports, our community-based study found that reduced daytime HRV is significantly associated with an increased risk of pro-atherogenic effects. We propose several possible explanations for the relationship between ANS dysfunction and ICAS. Firstly, low HRV is often indicative of dysfunction in the ANS, which can disrupt the beat-to-beat regulation of cerebral blood flow, leading to insufficient oxygen supply to brain tissue and promoting the development of ICAS [49]. Moreover, the relationship between HRV and ICAS may be influenced by other vascular risk factors, such as hypertension. Sympathetic overactivity caused by ANS dysfunction was correlated with hypertension due to restricted vasodilation, resulting in increased ambulatory blood pressure and reduced blood flow, thereby increasing the risk of ICAS [50]. Consistent with previous reports, in our study, we found hypertension as a covariate in our models to address any potential confounding effects of hypertension on the relationship between HRV and ICAS [51,52]. Lastly, evidence suggests that endothelial cells play an important role in regulating vascular homeostasis [53] and that endothelial dysfunction is an early marker for atherosclerosis [54]. Suppressed heart rate suggests impaired autonomic balance, induces inflammation, and disrupts endothelial homeostasis, promoting the production of vasoconstrictors (e.g., endothelin and angiotensin II), which activate macrophages and vascular smooth muscle cells, ultimately contributing to plaque formation in intracranial atherosclerosis [54,55]. Therefore, lower HRV may be an early predictor autonomic dysfunction, and is associated with impaired vascular health.

In addition, we have found that atrial hypoxia, an indicator of myocardial oxygen metabolism function, is a strong risk predictor of increased risk presence of ICAS, but the primary mechanisms are intricate and interconnected. The possible causes may include myocardial metabolic disturbances, hemodynamic changes due to cerebral vascular remodeling and arterial stiffness, and oxidative stress effects. Metabolic disturbances in the heart can elevate reactive oxygen species (ROS) and impair antioxidant defenses, thereby inducing oxidative stress that disrupts endothelial function. This leads to endothelial dysfunction and the release of endothelin-1 (ET-1), a potent vasoconstrictor released by endothelial cells. ET-1 may significantly augment the hemodynamic significance of coronary stenoses, thereby exacerbating myocardial ischemia [56]. Furthermore, structural remodeling of vascular dimensions affects cerebral blood flow distribution and altered flow patterns can trigger inflammation and exacerbate endothelial dysfunction, facilitating the progression of ICAS [57].

From another perspective, we built upon previous work by utilizing 3 min HRV monitoring to efficiently assess the association between HRV and various imaging features of atherosclerotic plaques, aiming to identify high-risk patients to be shortlisted for more complex stratifications. Based on prior studies and our previous histopathologic evidence, an eccentric thickening lesion was frequently used as a criterion for identifying ICAS and may be associated with ischemic events [58,59,60]. Our current investigation, however, failed to indicate the association between any intracranial atherosclerotic plaque imaging features and HRV. This may be due to the relatively small sample size and compensatory mechanisms such as collateral circulation that may potentially mitigate the impact of damage to the cortical or subcortical structures or of the neural pathways known to regulate the cardiovascular autonomic system. In addition, despite the absence of evidence supporting the correlation between frequency domain measures (HF, LF, and LF/HF) and ICAS, this lack of association could potentially be attributed to the limitations of short-term ECG recordings, which restrict the applicability of power spectral analysis to accurately capture LF and HF components [61]. This study is an observational study conducted at the HK community sample. However, robust recruitment strategies of random selection of Chinese participants from different communities in Hong Kong minimized potential selection bias. Moreover, clinical information on vascular risk factors, including hypertension and diabetes mellitus, was derived from standardized self-reported questionnaires rather than laboratory verification. While this method may introduce recall bias, it enables efficient, cost-effective data collection in community-based cohorts. Self-reported health information from questionnaires has been widely used in epidemiological research and can provide reasonable estimates of vascular risk profiles when combined with the standardized questionnaire and trained interviewer. Additionally, compared with conventional HRV analyses, ECG-DM (3 min recording) features a shorter collection time and simpler data processing. However, this novel short-term ECG-DM technology is highly sensitive, such that even minor signal abnormalities can markedly influence the resulting indicators used for comprehensive physiological analysis. The selection of an appropriate heart rate data collection method should consider the specific research design or clinical background. For evaluations of long-term dynamic autonomic function and malignant ventricular arrhythmias following myocardial infarction, conventional long-term HRV analysis is preferred, whereas for analyzing data over shorter durations to provide early warning of potential instantaneous myocardial hypoxic metabolic injury and autonomic function disorder, the short-term method is generally sufficient. Furthermore, we did not include antihypertensive and other cardiovascular medications in the confounder analysis due to the relatively small sample size and the limited number of participants receiving such treatments. Moreover, we excluded antihypertensive and other cardiovascular medications from the adjusted models because substantial collinearity with key covariates (e.g., hypertension), which could destabilize the models. Last but not least, given that this study is cross-sectional, it is difficult to imply a direct predictive value of lower HRV for the presence of intracranial atherosclerosis. Longitudinal studies are needed to verify these findings and determine the exact relationship. Further prospective studies with larger, multi-ethnic samples are warranted to validate the clinical application of 3 min ECG-DM cardiac function monitoring for identifying ICAS severity and related clinical outcomes, and to incorporate in-depth analyses that treat SDNN as a continuous variable to evaluate the robustness of the dose–response relationship.

ECG dispersion mapping is a state-of-the-art developmental technique that enables noninvasive assessment of myocardial pathology and contributes to pre-nosological diagnosis [62]. In the present study, 3 min ECG-DM was employed as a novel approach for the early detection of ischemic myocardial metabolic impairment and autonomic dysfunction associated with brain vascular health. Compared with direct “beat-to-beat” long-duration measurement of ECG micro-alternations, this method is much more sensitive and permits assessment under resting conditions. Notably, the 3 min acquisition aligns with the short-term HRV consensus: although a 5 min resting segment is the conventional standard, methodological studies support reliable estimation of time-domain indices from 2 to 5 min recordings under resting conditions [16,17]. To the best of our knowledge, the 3 min ECG dispersion mapping (ECG-DM) protocol has been validated primarily in human studies to date—across community-based and clinical cohorts—focusing on autonomic function and myocardial metabolic assessment. Systematic experimental validation in animal models is still lacking. In the future, rigorous, controlled animal studies are warranted to elucidate the electrophysiological mechanisms and to establish the biological correlations of 3 min ECG-DM parameters under diverse physiological and pathological conditions. Although other techniques may outperform ECG-DM in diagnosing cardiac disease, potential asymptomatic disturbances in the autonomic regulatory balance of the brain–heart axis and transient hypoxia-related metabolic impairment of the myocardium are more likely driven by rapid changes in cerebral hemodynamics and tissue oxygenation than by disease severity per se. Distinct from many diagnostic modalities and clinical judgment, the 3 min ECG-DM protocol provides an objective, comprehensive, and highly efficient assessment, is inexpensive to perform, and requires no specialized expertise for interpretation.

## 5. Conclusions

The study’s findings suggest that lower HRV may serve as a valuable indicator of cardiac dysfunction and is associated with the presence of intracranial atherosclerosis. These findings may improve clinical risk management and guide the prevention strategies for cerebrovascular events. Further prospective evidence is needed to validate the causal relationships and underlying mechanisms between HRV and cSVD.

## Figures and Tables

**Table 1:** Demographic and clinical characteristics of subjects with or without ICAS.

Characteristics	Total (n = 272)	Adults Without ICAS (n = 120)	Adults with ICAS (n = 152)	*p*-Value
Age, y, mean ± SD	63.4 ± 6.8	62.4 ± 6.7	64.1 ± 6.8	0.041
Sex, male, n (%)	118 (43.4)	46 (38.3)	72 (47.4)	0.135
SBP, mmHg, (mean ± SD)	129.9 ± 17.1	126.4 ± 17.5	132.7 ± 16.3	0.003
DBP, mmHg, (mean ± SD)	79.9 ± 10.5	77.8 ± 10.7	81.5 ± 10.1	0.005
Body mass index ≥25 kg/m^2^, n (%)	87 (32.0)	27 (22.5)	60 (39.5)	0.003
Hypertension	80 (29.5)	21 (17.5)	59(38.8)	<0.001
Diabetes mellitus	33 (12.2)	13 (10.8)	20 (13.2)	0.560
Coronary artery disease	17 (6.3)	4 (3.3)	13 (8.6)	0.077
Hyperlipidemia	104 (38.4)	32 (26.7)	72 (47.4)	<0.001
Statins	84 (30.9)	25 (21.0)	59 (38.8)	0.002
Antiplatelet	13 (4.8)	4 (3.4)	9 (5.9)	0.328
Anticoagulant	31 (11.4)	11 (9.2)	20 (13.2)	0.315
Antihypertensives	73 (26.8)	18 (15.1)	55 (36.2)	<0.001
Antidiabetics	16 (5.9)	6 (5.0)	10 (6.6)	0.594
Smoker	14 (5.2)	5 (4.2)	9 (5.9)	0.516
Alcohol Drinking	20 (7.4)	9 (7.5)	11 (7.2)	0.934

SBP indicates systolic blood pressure; DBP, diastolic blood pressure; WMH, white matter hyperintensity; SD, standard deviation.

**Table 2:** The comparison of ECG characteristics between the participants with and without ICAS.

Characteristics	Total (n = 272)	Adults Without ICAS (n = 120)	Adults with ICAS (n = 152)	*p*-Value
Abnormal, n (%)	102 (37.5)	45 (37.5)	57 (37.7)	0.967
Heart rate, bmp	72.2 ± 10.4	71.9 ± 11.2	72.4 ± 9.8	0.740
MMI, mean ± SD	19.1 ± 8.6	19.8 ± 10.4	18.4 ± 6.9	0.193
**Atrial deviation, n (%)**	175 (64.3)	69 (57.5)	106 (70.2)	0.030
Ventricular deviation, n (%)	235 (86.3)	103 (85.8)	132 (87.4)	0.703
Power HF, ms^2^ median (IQR)	154.9 (67.4, 319.8)	161.5 (75.0, 317.8)	154.6 (64.2, 320.6)	0.345
Power LF, ms^2^ median (IQR)	100.5 (47.0, 235.2)	96.3 (52.7, 194.8)	107.7 (43.7, 270.3)	0.326
LF/HF < 1, n (%)	168 (61.7)	71 (59.2)	97 (64.2)	0.393
SDNN, abnormal, n (%)	94 (34.5)	34 (28.3)	60 (39.7)	0.040

ECG, electrocardiogram; MMI, myocardial micro-alteration index; SDNN, standard deviation of normal-to-normal intervals; power HF (ms^2^), high frequency; power LF (ms^2^), low frequency (ms^2^); power ratio (LF/HF), the low to high frequency.

**Table 3:** The comparison of characteristics of subjects among different SDNN values.

Characteristics	Group 1 (n = 90)	Group 2 (n = 91)	Group 3 (n = 91)	*p*-Value
Age, y, mean ± SD	65.0 ± 5.9	63.3 ± 6.6	62.0 ± 7.4 ^ac^	0.010
Sex, male, n (%)	40 (44.4)	38 (41.8)	40 (44.4)	0.915
SBP, mmHg, mean ± SD	128.8 ± 17.7	131.5 ± 16.9	128.9 ± 16.4	0.487
DBP, mmHg, mean ± SD	79.1 ± 9.4	80.9 ± 11.1	79.3 ± 10.7	0.432
Body mass index ≥25 kg/m^2^, n (%)	26 (28.9)	31 (34.1)	30 (33.3)	0.723
Hypertension	31 (34.4)	32 (35.2) ^bc^	17 (18.9) ^ac^	0.026
Diabetes mellitus	14 (15.6)	11 (12.1)	8 (8.9)	0.392
Coronary artery disease	7 (7.8)	5 (5.5)	5 (5.6)	0.771
Hyperlipidemia	36 (34.6)	33 (36.3)	35 (38.9)	0.868
The presence of ICAS	50 (55.5)	44 (48.3) ^ab^	45 (49.4) ^ac^	0.032
Plaque burden%, mean ± SD	47.4 ± 3.7	38.5 ± 3.8	44.8 ±3.9	0.243
Luminal stenosis, mean ± SD	23.0 ± 19.8	17.1 ± 19.9	17.4 ± 20.1	0.121
Irregular surface, n (%)	21 (23.3)	22 (24.2)	21 (23.3)	0.933
Diffuse lesion, n (%)	35 (38.9)	38 (41.8)	36 (40.0)	0.757
Eccentric lesion, n (%)	40 (44.4)	38 (41.8)	41 (45.6)	0.869
Positive remodeling, n (%)	33 (36.7)	28 (30.8)	33 (36.7)	0.629

The *p*-values in the fifth column represent the level of significance across the three study groups. Tertiles of the SDNN (standard deviation of normal-to-normal intervals) were categorized into three groups for analysis. The groups were defined as follows: Group 1 includes SDNN values from 0 to 20.75 ms; Group 2 includes SDNN values from 20.76 to 29.64 ms; Group 3 consists of SDNN values greater than or equal to 29.65 ms. ^ac^, Group 1 vs. Group 3, *p*-value < 0.05; ^ab^, Group 1 vs. Group 2, *p*-value < 0.05; ^bc^, Group 2 vs. Group 3, *p*-value < 0.05.

**Table 4:** Multiple logistic regression models on the correlation between HRV and the burden of ICAS.

	Intracranial Large Artery Lesion, Odds Ratio (95% CI)	*p*-Value
**Characteristics**		
SDNN deviation	1.40 (1.01–1.94)	**0.044**
Atrial hypoxia	1.74 (1.05–2.87)	**0.031**
Model 1 Adjusted for confounding factors (age, gender)		
SDNN deviation	1.57 (1.12–2.21)	**0.009**
Atrial hypoxia	1.72 (1.03–2.85)	**0.038**
Model 2 Adjusted for confounding factors (age, WMH burden, gender, hypertension, BMI, hyperlipidemia)		
SDNN deviation	1.55 (1.10–2.18)	**0.012**
Atrial hypoxia	1.85 (1.10–3.14)	**0.022**

*p* < 0.05; OR, odds ratio; 95% CI, 95% confidence interval; SDNN analyzed in regression model as a binary categorical variable: normal (17.9–40.1 ms) vs. abnormal (outside this range).

**Table 5:** The inter-observer reliability of analyzing the presence of intracranial atherosclerosis (ICAS) and other plaque imaging features.

	Rater 1	Rater 2	Inter-Observer Agreement Coefficient (95% CI)
Presence of ICAS (MCA, BA, VA); n, %	127 (55.9)	123 (54.2)	0.86 (0.793–0.926)
Presence of ICAS (MCA, BA, VA, ACA, PCA, ICA); n, %	152 (66.9%)	144 (63.4)	0.83 (0.753–0.906)
Plaque eccentricity; n, %	102 (45%)	86 (37.9%)	0.77 (0.692–0.847)
Plaque irregular surface; n, %	51 (22.5)	45 (20.0%)	0.82 (0.727–0.912)
Plaque diffuse; n, %	94 (41.4%)	78 (34.4%)	0.71 (0.631–0.797)

## Data Availability

The datasets generated and/or analyzed during the current study are available from the corresponding author (Michael Ying) on reasonable request.

## References

[1] Lange M.C., Ribas G., Scavasine V., Ducci R.D.-P., Mendes D.C., Zétola V.d.H.F., Cabral N., Rundek T. (2018). Stroke recurrence in the different subtypes of ischemic stroke. The importance of the intracranial disease. Arq. Neuropsiquiatr..

[2] Wang Y., Zhao X., Liu L., Soo Y.O., Pu Y., Pan Y., Wang Y., Zou X., Leung T.W., Cai Y. (2014). Prevalence and outcomes of symptomatic intracranial large artery stenoses and occlusions in China: The Chinese Intracranial Atherosclerosis (CICAS) Study. Stroke.

[3] Suri M.F.K., Qiao Y., Ma X., Guallar E., Zhou J., Zhang Y., Liu L., Chu H., Qureshi A.I., Alonso A. (2016). Prevalence of intracranial atherosclerotic stenosis using high-resolution magnetic resonance angiography in the general population: The atherosclerosis risk in communities study. Stroke.

[4] Del Brutto O.H., Mera R.M., Lama J., Zambrano M., Del Brutto V.J. (2015). Intracranial arterial stenosis in Ecuadorian Natives/Mestizos. A population-based study in older adults (The Atahualpa Project). Arch. Gerontol. Geriatr..

[5] Li S., Tang M., Zhang D., Han F., Zhou L., Yao M., Li M., Cui L., Zhang S., Peng B. (2023). The prevalence and prognosis of asymptomatic intracranial atherosclerosis in a community-based population: Results based on high-resolution magnetic resonance imaging. Eur. J. Neurol..

[6] Sun Q., Wang Q., Wang X., Ji X., Sang S., Shao S., Zhao Y., Xiang Y., Xue Y., Li J. (2020). Prevalence and cardiovascular risk factors of asymptomatic intracranial arterial stenosis: The Kongcun Town Study in Shandong, China. Eur. J. Neurol..

[7] Bang O.Y., Kim J.W., Lee J.H., Lee M.A., Lee P.H., Joo I.S., Huh K. (2005). Association of the metabolic syndrome with intracranial atherosclerotic stroke. Neurology.

[8] Feldmann E., Daneault N., Kwan E., Ho K.J., Pessin M.S., Langenberg P., Caplan L.R. (1990). Chinese-white differences in the distribution of occlusive cerebrovascular disease. Neurology.

[9] Kim S.J., Schneider D.J., Feldmann E., Liebeskind D.S. (2022). Intracranial atherosclerosis: Review of imaging features and advances in diagnostics. Int. J. Stroke.

[10] Kurtzke J.F. (2012). Epidemiology of Cerebrovascular Disease.

[11] Qureshi A.I., Caplan L.R. (2014). Intracranial atherosclerosis. Lancet.

[12] McLaren A., Kerr S., Allan L., Steen I.N., Ballard C., Allen J., Murray A., Kenny R.A. (2005). Autonomic function is impaired in elderly stroke survivors. Stroke.

[13] Esler M. (2000). The sympathetic system and hypertension. Am. J. Hypertens.

[14] Hamner J., Tan C.O., Lee K., Cohen M.A., Taylor J.A. (2010). Sympathetic control of the cerebral vasculature in humans. Stroke.

[15] Zhang R., Zuckerman J.H., Iwasaki K., Wilson T.E., Crandall C.G., Levine B.D. (2002). Autonomic neural control of dynamic cerebral autoregulation in humans. Circulation.

[16] Essibayi M.A., Toma A., Mowrey W., Qin J., Hamad M., Ryvlin J., Holland R., Fluss R., Altschul D., Lin L.-M. (2025). Heart rate and heart rate variability during diagnostic and interventional neuroendovascular procedures. Interv. Neuroradiol..

[17] Task Force of the European Society of Cardiology the North American Society of Pacing Electrophysiology (1996). Heart rate variability: Standards of measurement, physiological interpretation, and clinical use. Circulation.

[18] Dekker J.M., Crow R.S., Folsom A.R., Hannan P.J., Liao D., Swenne C.A., Schouten E.G. (2000). Low heart rate variability in a 2-minute rhythm strip predicts risk of coronary heart disease and mortality from several causes: The ARIC Study. Atherosclerosis Risk In Communities. Circulation.

[19] Nelde A., Krumm L., Arafat S., Hotter B., Nolte C.H., Scheitz J.F., Klammer M.G., Krämer M., Scheib F., Endres M. (2024). Machine learning using multimodal and autonomic nervous system parameters predicts clinically apparent stroke-associated pneumonia in a development and testing study. J. Neurol..

[20] Qiu Q., Song W., Zhou X., Yu Z., Wang M., Hao H., Pan D., Luo X. (2022). Heart rate variability is associated with cerebral small vessel disease in patients with diabetes. Front. Neurol..

[21] Wang H., Jiang J., Zhou G., Zhang Y. (2025). The Roles of Heart Rate Variability in Cerebral Stroke. Neuropsychiatr. Dis. Treat..

[22] Makikallio A.M., Makikallio T.H., Korpelainen J.T., Sotaniemi K.A., Huikuri H.V., Myllyla V.V. (2004). Heart rate dynamics predict poststroke mortality. Neurology.

[23] Fyfe-Johnson A.L., Muller C.J., Alonso A., Folsom A.R., Gottesman R.F., Rosamond W.D., Whitsel E.A., Agarwal S.K., MacLehose R.F. (2016). Heart rate variability and incident stroke: The atherosclerosis risk in communities study. Stroke.

[24] Pramanda A.N., Farabi F., Prameswari H.S., Achmad C., Tiksnadi B.B. (2025). Myocardial-alternation index (MMI) is correlated with soluble suppression of tumorigenecity-2 (sST2) in patients with ischemic cardiomyopathy. Egypt. Heart J..

[25] Tiksnadi B.B., Putra A.P., Ikhsani R., Tarsidin N.F. (2024). Myocardial micro-alternation index (MMI) does not associate with the ischemic response in coronary artery disease patients. Eur. J. Prev. Cardiol..

[26] Watanabe E., Kiyono K., Hayano J., Yamamoto Y., Inamasu J., Yamamoto M., Ichikawa T., Sobue Y., Harada M., Ozaki Y. (2015). Multiscale Entropy of the Heart Rate Variability for the Prediction of an Ischemic Stroke in Patients with Permanent Atrial Fibrillation. PLoS ONE.

[27] Kellett J., Rasool S., McLoughlin B. (2012). Prediction of mortality 1 year after hospital admission. QJM Int. J. Med..

[28] Li J., Yang W.-J., Zheng L., Du H., Chu W.C.-W., Leung T.W.-H., Chen X.-Y. (2021). Vertebrobasilar Junction Angle Over 90°: A Potential Imaging Marker Associated With Vertebrobasilar Atherosclerosis. Front. Neurosci..

[29] Wang M., Wu F., Yang Y., Miao H., Fan Z., Ji X., Li D., Guo X., Yang Q. (2018). Quantitative assessment of symptomatic intracranial atherosclerosis and lenticulostriate arteries in recent stroke patients using whole-brain high-resolution cardiovascular magnetic resonance imaging. J. Cardiovasc. Magn. Reson..

[30] Yang W.-J., Abrigo J., Soo Y.O.-Y., Wong S., Wong K.-S., Leung T.W.-H., Chu W.C.-W., Chen X.-Y. (2020). Regression of Plaque Enhancement Within Symptomatic Middle Cerebral Artery Atherosclerosis: A High-Resolution MRI Study. Front. Neurol..

[31] Zheng L., Li J., Yang W., Lam H.-C.C., Wong K.L., Chu W., Leung T.W.H., Chen X. (2023). Patterns and Implications of Intracranial Atherosclerosis in Anterior and Posterior Circulation Identified by High-Resolution Vessel Wall Imaging. Cerebrovasc. Dis..

[32] Dieleman N., Yang W., Abrigo J.M., Chu W.C.W., van der Kolk A.G., Siero J.C., Wong K.S., Hendrikse J., Chen X.Y. (2016). Magnetic Resonance Imaging of Plaque Morphology, Burden, and Distribution in Patients with Symptomatic Middle Cerebral Artery Stenosis. Stroke.

[33] Dieleman N., Yang W., van der Kolk A.G., Abrigo J., Lee K.L., Chu W.C.W., Zwanenburg J.J.M., Siero J.C.W., Wong K.S., Hendrikse J. (2016). Qualitative Evaluation of a High-Resolution 3D Multi-Sequence Intracranial Vessel Wall Protocol at 3 Tesla MRI. PLoS ONE.

[34] Yang W.-J., Wasserman B.A., Zheng L., Huang Z.-Q., Li J., Abrigo J., Wong S.S.-M., Ying M.T.-C., Chu W.C.-W., Wong L.K.-S. (2021). Understanding the Clinical Implications of Intracranial Arterial Calcification Using Brain CT and Vessel Wall Imaging. Front. Neurol..

[35] Li J., Zheng L., Yang W.-J., Sze-To C.-Y., Leung T.W.-H., Chen X.-Y. (2020). Plaque Wall Distribution Pattern of the Atherosclerotic Middle Cerebral Artery Associates With the Circle of Willis Completeness. Front. Neurol..

[36] Yang W.J., Fisher M., Zheng L., Niu C.B., Paganini-Hill A., Zhao H.L., Xu Y., Wong K.S., Ng H.K., Chen X.Y. (2017). Histological Characteristics of Intracranial Atherosclerosis in a Chinese Population: A Postmortem Study. Front. Neurol..

[37] Qiao Y., Anwar Z., Intrapiromkul J., Liu L., Zeiler S.R., Leigh R., Zhang Y., Guallar E., Wasserman B.A. (2016). Patterns and Implications of Intracranial Arterial Remodeling in Stroke Patients. Stroke.

[38] Bai X., Fan P., Li Z., Mossa-Basha M., Ju Y., Zhao X., Kong Q., Pei X., Zhang X., Sui B. (2024). Evaluating Middle Cerebral Artery Plaque Characteristics and Lenticulostriate Artery Morphology Associated With Subcortical Infarctions at 7T MRI. J. Magn. Reson Imaging.

[39] Tao L., Li X.Q., Hou X.W., Yang B.Q., Xia C., Ntaios G., Chen H.S. (2021). Intracranial Atherosclerotic Plaque as a Potential Cause of Embolic Stroke of Undetermined Source. J. Am. Coll. Cardiol..

[40] Alkan O., Kizilkilic O., Yildirim T., Atalay H. (2009). Intracranial cerebral artery stenosis with associated coronary artery and extracranial carotid artery stenosis in Turkish patients. Eur. J. Radiol..

[41] Samuels O.B., Joseph G.J., Lynn M.J., Smith H.A., Chimowitz M.I. (2000). A standardized method for measuring intracranial arterial stenosis. AJNR Am. J. Neuroradiol..

[42] Yasaka M., Yamaguchi T., Shichiri M. (1993). Distribution of atherosclerosis and risk factors in atherothrombotic occlusion. Stroke.

[43] Dong J.G. (2016). The role of heart rate variability in sports physiology. Exp. Ther. Med..

[44] Shaffer F., Ginsberg J.P. (2017). An Overview of Heart Rate Variability Metrics and Norms. Front. Public Health.

[45] Nakanishi K., Jin Z., Homma S., Elkind M.S., Rundek T., Lee S.C., Tugcu A., Yoshita M., DeCarli C., Wright C.B. (2018). Association Between Heart Rate and Subclinical Cerebrovascular Disease in the Elderly. Stroke.

[46] Korpelainen J.T., A Sotaniemi K., Suominen K., Tolonen U., Myllylä V.V. (1994). Cardiovascular autonomic reflexes in brain infarction. Stroke.

[47] Lees T., Shad-Kaneez F., Simpson A.M., Nassif N.T., Lin Y., Lal S. (2018). Heart rate variability as a biomarker for predicting stroke, post-stroke complications and functionality. Biomark. Insights.

[48] Whelton S.P., Blankstein R., Al-Mallah M.H., Lima J.A., Bluemke D.A., Hundley W.G., Polak J.F., Blumenthal R.S., Nasir K., Blaha M.J. (2013). Association of resting heart rate with carotid and aortic arterial stiffness: Multi-ethnic study of atherosclerosis. Hypertension.

[49] Arenillas J.F. (2015). Intracranial atherosclerosis and inflammation: Lessons from the East and the West. Brain Circ..

[50] Koichubekov B., Sorokina M., Laryushina Y., Turgunova L., Korshukov I. (2018). Nonlinear analyses of heart rate variability in hypertension. Ann. Cardiol. Angeiol..

[51] Ebinger J.E., Driver M.P., Huang T.Y., Magraner J., Botting P.G., Wang M., Chen P.-S., Bello N.A., Ouyang D., Theurer J. (2024). Blood pressure variability supersedes heart rate variability as a real-world measure of dementia risk. Sci. Rep..

[52] Takahashi M.K.N., Paradela R.S., Grinberg L.T., Leite R.E.P., Farias-Itao D.S., Paes V.R., Braga M.E., Naslavsky M.S., Zatz M., Jacob-Filho W. (2025). Hypertension may associate with cerebral small vessel disease and infarcts through the pathway of intracranial atherosclerosis. Neurobiol. Aging.

[53] Kinlay S., Libby P., Ganz P. (2001). Endothelial function and coronary artery disease. Curr. Opin. Lipidol..

[54] Davignon J., Ganz P. (2004). Role of endothelial dysfunction in atherosclerosis. Circulation.

[55] Drexler H. (1998). Factors involved in the maintenance of endothelial function. Am. J. Cardiol..

[56] Kinlay S., Behrendt D., Wainstein M., Beltrame J., Fang J.C., Creager M.A., Selwyn A.P., Ganz P. (2001). Role of endothelin-1 in the active constriction of human atherosclerotic coronary arteries. Circulation.

[57] Chen L.H., Spagnolo-Allende A., Yang D., Qiao Y., Gutierrez J. (2024). Epidemiology, Pathophysiology, and Imaging of Atherosclerotic Intracranial Disease. Stroke.

[58] Ahn S.-H., Lee J., Kim Y.-J., Kwon S.U., Lee D., Jung S.-C., Kang D.-W., Kim J.S. (2015). Isolated MCA disease in patients without significant atherosclerotic risk factors: A high-resolution magnetic resonance imaging study. Stroke.

[59] Ohara T., Toyoda K., Otsubo R., Nagatsuka K., Kubota Y., Yasaka M., Naritomi H., Minematsu K. (2008). Eccentric stenosis of the carotid artery associated with ipsilateral cerebrovascular events. AJNR Am. J. Neuroradiol..

[60] Swartz R.H., Bhuta S.S., Farb R.I., Agid R., Willinsky R.A., Terbrugge K.G., Butany J., Wasserman B.A., Johnstone D.M., Silver F.L. (2009). Intracranial arterial wall imaging using high-resolution 3-tesla contrast-enhanced MRI. Neurology.

[61] Korpelainen J.T., Sotaniemi K.A., Huikuri H.V., Myllylä V.V. (1996). Abnormal heart rate variability as a manifestation of autonomic dysfunction in hemispheric brain infarction. Stroke.

[62] Esina E., Zuikova A., Dobrynina I., Lyutov V., Tsygan V. (2021). ECG Dispersion Mapping in Preclinical Diagnosis of Cardiovascular Diseases. Sovrem Tekhnologii Med..

